# Optimization of pacing parameters to entrain slow wave activity in the pig jejunum

**DOI:** 10.1038/s41598-024-56256-2

**Published:** 2024-03-13

**Authors:** Nipuni D. Nagahawatte, Recep Avci, Niranchan Paskaranandavadivel, Leo K. Cheng

**Affiliations:** 1 Auckland Bioengineering Institute, University of Auckland, Private Bag 92019, Auckland, 1142 New Zealand; 2 Department of Surgery, Vanderbilt University, Nashville, TN USA; 3 Riddet Institute Centre of Research Excellence, Palmerston North, New Zealand

**Keywords:** Physiology, Gastroenterology, Engineering

## Abstract

Pacing has been proposed as a therapy to restore function in motility disorders associated with electrical dysrhythmias. The spatial response of bioelectrical activity in the small intestine to pacing is poorly understood due to a lack of high-resolution investigations. This study systematically varied pacing parameters to determine the optimal settings for the spatial entrainment of slow wave activity in the jejunum. An electrode array was developed to allow simultaneous pacing and high-resolution mapping of the small intestine. Pacing parameters including pulse-width (50, 100 ms), pulse-amplitude (2, 4, 8 mA) and pacing electrode orientation (antegrade, retrograde, circumferential) were systematically varied and applied to the jejunum (n = 15 pigs). Pulse-amplitudes of 4 mA (*p* = 0.012) and 8 mA (*p* = 0.002) were more effective than 2 mA in achieving spatial entrainment while pulse-widths of 50 ms and 100 ms had comparable effects (*p* = 0.125). A pulse-width of 100 ms and a pulse-amplitude of 4 mA were determined to be most effective for slow wave entrainment when paced in the antegrade or circumferential direction with a success rate of greater than 75%. These settings can be applied in chronic studies to evaluate the long-term efficacy of pacing, a critical aspect in determining its therapeutic potential.

## Introduction

Pacing applies high-energy electrical pulses at a frequency similar to intrinsic activity with the aim of enhancing or restoring disordered bioelectrical activity and function^1^. Cardiac pacemakers deliver extrinsic pulses that aim to correct cardiac arrythmias and are used to treat fatal conditions such as bradycardia, heart failure and syncope^2,3^. Significant research has also been conducted to investigate the potential of this technique in treating illnesses associated with other organs of the body including the stomach^4,5^.

Slow waves are a critical component for coordinating the motility patterns of the gastrointestinal (GI) tract^6^. Abnormal initiation and conduction of slow waves have been previously linked to functional motility disorders such as gastroparesis and chronic nausea^7–9^. Gastric pacing seeks to modulate the intrinsic bioelectrical rhythm of the stomach and motility patterns. This is contrary to high-frequency stimulation which has been shown to primarily provide symptom relief, but does not alter slow wave patterns^10–12^ or gastric emptying^13,14^. Therefore, this study focused on optimizing pacing parameters to modulate slow wave patterns. Gastric pacing has previously shown success in entraining both temporal and spatial characteristics of slow waves and therefore, is a promising therapy for motility disorders^15–17^.

Small intestine pacing has also been investigated for its clinical potential and has reported success in modulating the electrical and motility responses alongside absorption rates^18–20^. Electrical activity has been modulated to normalize dysrhythmias in intact and transected intestinal segments^20–22^. In addition, motility responses associated with rate of contractions and emptying, as well as direction of content flow have been regulated^19,23,24^, along with rates of nutrient absorption^19,21,25^. These findings are highly relevant in treating multi-factorial conditions such as prolonged post-operative ileus and short bowel syndrome, which can be challenging to treat with conventional approaches^26,27^. A recent systematic review revealed pacing orientation plays a key role in enhancing and inhibiting these responses, which highlights the critical role of individual pacing parameters and the need for their systematic evaluation^18^.

Historically, GI pacing response has been evaluated using low-resolution mapping techniques, but recent gastric pacing studies have utilized high-resolution techniques such as electrical and optical mapping frameworks^17,28^. With the development of high-resolution surface-contact electrode arrays^17,29,30^, and miniaturized wireless pacemakers^31,32^, the understanding of the gastric pacing response has been immensely expanded. However, pacing response of the small intestine is yet to be investigated using high-resolution techniques to determine optimal settings. The need for appropriate high-resolution electrode arrays and implantable devices has been previously highlighted^18^, and a novel framework which enables high-resolution electrical mapping was recently introduced^33^.

This study evaluated the efficacy of the pacing response in the jejunum in high-resolution by systematically varying the pacing parameters. A novel high-resolution framework developed for simultaneous pacing and mapping of the small intestine response was used, along with signal-processing frameworks customized for pacing recordings^33,34^. Slow wave response was quantitatively and qualitatively evaluated and histological examinations were conducted to assess for tissue damage.

## Methods

### Experimentation

#### Animal preparation

Ethical approval was granted by the University of Auckland Animal Ethics Committee and all methods were performed in accordance with the relevant guidelines and regulations. The study is reported in accordance with the ARRIVE guidelines.

Experiments were performed *in vivo* on 15 female crossbreed weaner pigs (weight = 40.9 ± 2.2 kg, 12–14 weeks old). The animal preparation, anesthesia, surgical access and monitoring were previously reported^33,35^. In brief, pigs were anesthetized by inducing Zoletil and maintained with isoflurane. A midline laparotomy was performed, and a proximal jejunal segment of approximately 20 cm in length was exteriorized with minimal handling. Recordings commenced less than 5 min after the laparotomy. At the conclusion of each study, the pigs were euthanized by injecting a bolus of sodium pentobarbital while still under anesthesia.

#### High-resolution mapping and pacing

**Figure 1:**
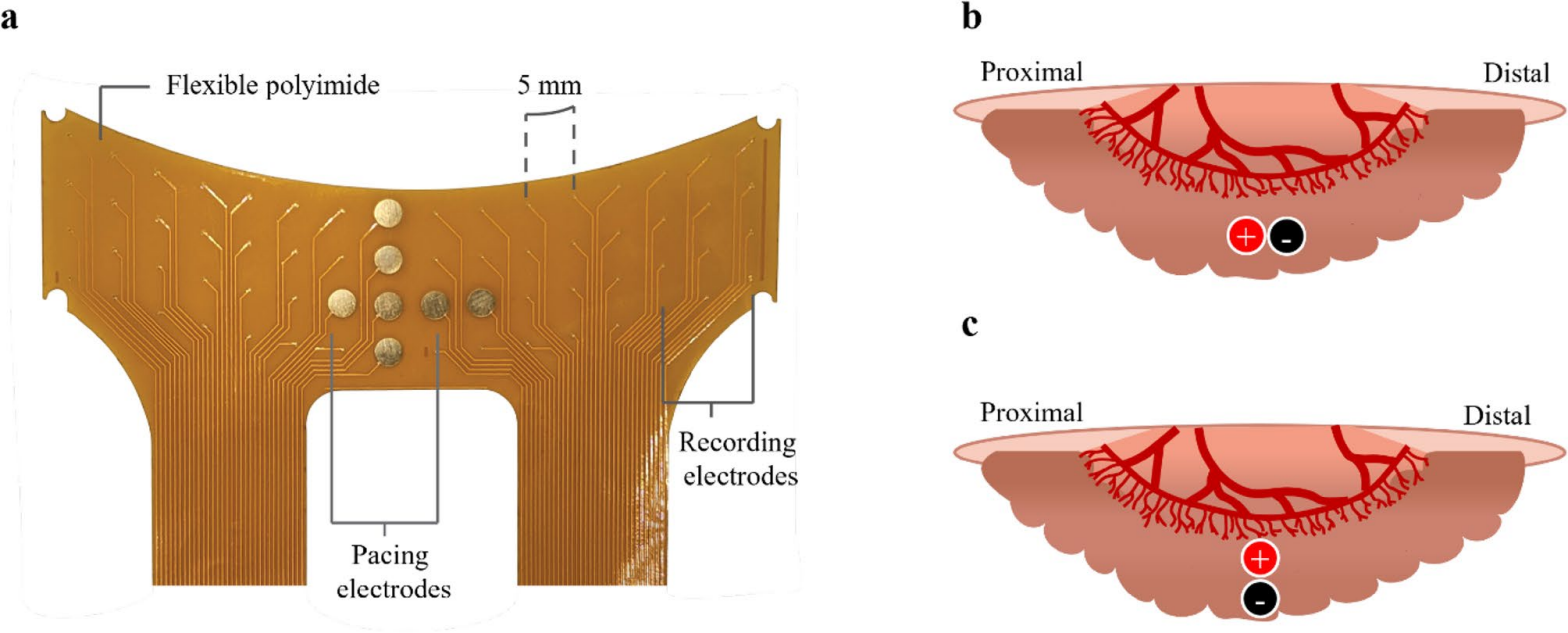
Small intestine pacing electrode and orientations. (**a**) Surface-contact electrode array used for simultaneous pacing and high-resolution mapping of the pacing response. (**b**) Longitudinal orientation of pacing electrodes enabled antegrade pacing. Retrograde pacing was achieved when the polarity was reversed such that the positive terminal was distal to the negative terminal. (**c**) The pacing electrodes were oriented along the circumferential axis of the intestine for circumferential pacing.

A surface-contact electrode array customized for simultaneous pacing and high-resolution mapping of the small intestine was used (see Fig. 1a). The specifications and the placement of the electrode arrays have been previously described^33^. In summary, each electrode array had a total of 64 electrodes with 5 mm spacing and gold contacts (57 electrodes had a diameter of 0.3 mm and 7 electrodes with a diameter of 3 mm). In each study, two of the larger 3 mm electrodes positioned at the center were selected for pacing based on the desired pacing orientation. The jejunal segment was sandwiched between 2 electrode arrays making contact with the top and bottom surfaces of the intestine. Plastic wrap (Glad, Clorox New Zealand Ltd., Auckland, New Zealand) was laid on top of the jejunum to minimize evaporation and cooling of the intestine.

Electrical activity from 126 channels (114 electrodes with 0.3 mm diameter and 12 electrodes with 3 mm diameter) was recorded at 512 Hz using an ActiveTwo signal acquisition system (BioSemi, Amsterdam, Netherlands) modified for passive recordings. Pacing was applied via 2 pacing electrodes (3 mm diameter) located on the bottom array by a DS8000 stimulator (World Precision Instruments, Inc., Sarasota, FL, USA).

Baseline activity was recorded for 2 min prior to pacing and the intrinsic slow wave frequency (IF) was estimated. Pacing was then applied for another 2 min at a frequency of 1.1 × IF, with the pulse-width (50 ms, 100 ms) and pulse-amplitude (2 mA, 4 mA, 8 mA) combinations randomized. Pacing was applied in the antegrade (see Fig. 1b), retrograde or circumferential (see Fig. 1c) direction as previously described in randomized order^33^. In 1980, Gladen et al., defined the terms antegrade and retrograde pacing which correspond to the orientation of pacing electrodes along the longitudinal axis. When the positive terminal (anode) was positioned anatomically proximal to the negative terminal (cathode), this configuration was termed antegrade pacing^36–38^. In contrast, placing the positive terminal anatomically distal to the negative terminal was classified as retrograde pacing. The intrinsic activity was recorded for a further 3 min after pacing was terminated and this duration is referred to as the post-pacing recording. Finally, the post-pacing recording was followed by a rest period of 5 min after which the same protocol was repeated until all 6 pacing settings were applied. The corresponding baseline, pacing and post-pacing segments of each pacing setting were collectively identified as a single session.

#### Histology

In order to determine whether pacing affected the integrity of the intestinal muscle layers, histology was performed on tissue from additional pacing sessions. At the end of all experiments, 2 pacing settings: (i) 4 mA, 100 ms and (ii) 8 mA, 100 ms were sequentially applied at a frequency of 1.1 × IF for 10 min approximately 10 cm apart. These settings correspond to the highest pulse-width combined with highest pulse-amplitudes. Sutures were applied adjacent to each pacing site on the tissue to mark the pacing location.

After euthanasia, an intestinal segment of approximately 30 cm was excised which contained the last 2 pacing locations along with control tissue. The segment was washed with warm saline and fixed in 20% natural buffered formalin solution. After allowing the tissues to fix for at least 48 h, the segment was extracted from the solution and cut into 6 tissue blocks of approximately 10 mm long through each of the pacing sites (a cutline for each setting) and control tissue (1 cutline)^33^. Each cutline provided 2 tissue blocks and all tissue blocks were embedded in paraffin. Tissue slices of 5 µm thick were sliced off each paraffin block and stained with hematoxylin and eosin. The stained slices were then imaged under a microscope to assess for tissue damage based on tissue structure, blood congestion, and swollen nuclei. The paced tissues were compared against control tissue slices.

### Data processing

#### Slow wave characterization

The raw signals were down sampled to 30 Hz for computational efficiency. The baseline wander of the acquired raw signals was estimated using a moving median filter (window width = 3 s) and smoothed using a Savitzky-Golay filter (polynomial order = 2; window = 2 s). The baseline drift was then removed by subtracting the estimated drift from the raw signal.

The large pacing artifacts superimposed on the slow wave recordings were detected using a Hampel outlier detection filter (number of neighbors = 15; number of standard deviations = 25) and removed using an autoregressive model^39^. The model parameters of the autoregressive model were defined using data accounting for a predefined window length of 0.5 s from both sides of each artifact segment.

The artifact removed signals were then visualized and analyzed using custom software (GEMS v3.0)^40^. To remove common mode noise, the median signal across all channels was computed and subtracted from each channel. Finally the ventilation noise was suppressed^41^. Post-filtering, the baseline segment and the post-pacing segment of each pacing setting were analyzed. The analysis of spatial entrainment was performed over 5 cycles post-pacing, as previous results have shown pacing response is maintained for approximately 5 cycles after pacing is stopped^33^. Activation times (AT) of individual slow wavefronts were automatically detected and clustered using validated algorithms^42,43^. The detected events were then manually reviewed to ensure accuracy.

The pacing response during each setting based on orientation, pulse-width and pulse-amplitude were evaluated to determine spatial entrainment of slow wave activity. Isochronal color maps were used to determine the slow wave propagation patterns where the initiation of a pacemaker propagation pattern confirmed spatial entrainment due to the paced activity. A pacemaker pattern was defined as the earliest activation of slow waves being observed at the center of the array, adjacent to the pacing electrodes and propagating towards the edges of the electrode array as rings of activations with a minimum of 80% coverage.

#### Energy quantification

To determine the energy applied at the pacing site corresponding to the histological analysis, the applied voltage was measured across the pacing electrodes for the 10-min duration (PicoScope 2000, Pico Technology, St Neots, UK). The captured pulses were approximately rectangular in shape and exhibited minimal capacitance effects. Therefore, a simplified ohmic calculation was performed to determine the energy applied during pacing. The measured voltage was averaged across the total duration, and thereby the tissue resistance between the 2 pacing electrodes was calculated using Eq. (1).

1 $$Resistance=\frac{Voltage}{Applied\,current}$$

where applied current was 4 mA or 8 mA for the 2 pacing sites. The total pulse duration (ON time) was determined using Eq. (2).

2 $$ON\,time=Paced\,duration\times Frequency\times Pulse{\text{-}}width$$

where paced duration was 10 min, frequency was 1.1 × IF (around 2.7 s) and pulse-width was 100 ms. Therefore, the total energy applied to the pacing site was calculated using Eq. (3).

3 $$Energy={Applied\,current}^{2}\times Resistance\times ON\, time$$

#### Pulse intensity

To determine the optimal pacing pulse-parameters for spatial entrainment, a pulse intensity was computed using Eq. (4)^29,44^.

4 $$Pulse\,intensity={Applied\,Current}^{2}\times Pulse{\text{-}}width$$

The energy corresponding for a particular pulse intensity can be interpreted by rearranging Eqs. (2–4). By scaling the pulse intensity with a factor accounting for paced duration, frequency and resistance, the energy can be calculated as given by Eq. (5).

5 $$Energy=Pulse\,intensity\times Resistance\times Paced\,duration\times Frequency$$

#### Statistical analysis

The data processing and statistical analyses were carried out using MATLAB R2022b (MathWorks, Natick, MA, USA), with all metrics being presented as the mean ± standard deviation. The normality of the data was determined using a Shapiro–Wilk test and a significance level of *P* < 0.05 was applied to all statistical tests.

The study used three-way analysis of variance (ANOVA) to examine the main and interaction effects of three pacing parameters (i.e., pulse-width, pulse-amplitude and pacing direction) on the rate of spatial entrainment, and Bonferroni's post hoc test was used for pairwise comparisons.

## Results

Slow wave activity was successfully spatially entrained in all 3 pacing directions and with all 6 combinations of pulse-amplitude and pulse-width. A total of 169 pacing studies with 1,014 slow wave cycles were analyzed from 15 pigs.

### Entrained slow wave propagation

**Figure 2:**
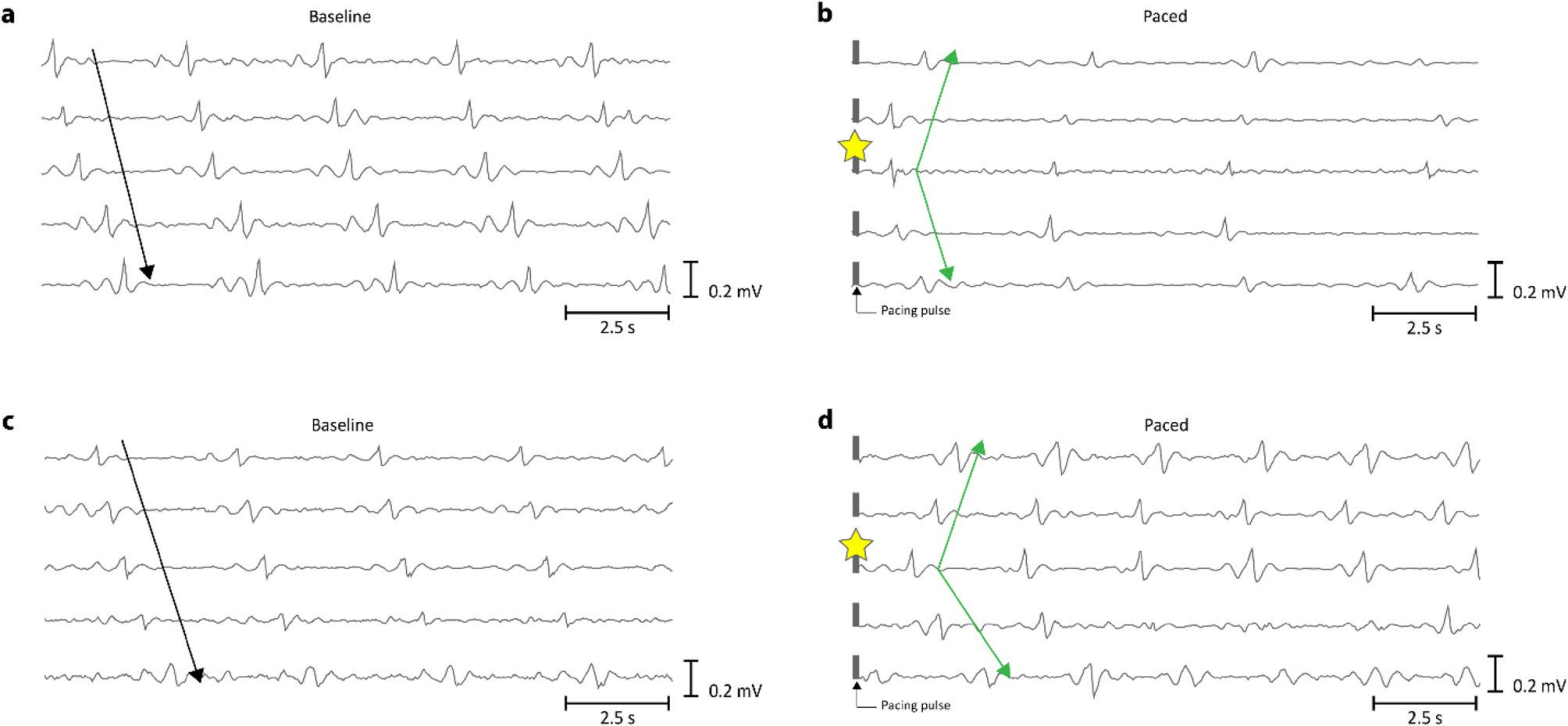
Signal traces of intrinsic and entrained slow wave activity. (**a**) and (**c**) are slow wave activity measured during baseline. (**b**) and (**d**) correspond to the post-pacing activity of (**a**) and (**c**) in order. The electrode located closest to the pacing electrodes is identified by a yellow star and was the site of the earliest activation indicating the origin of a pacemaker propagation pattern.

Two representative cases of slow wave activity entrained by pacing are shown in Fig. 2. Pacing was applied with an amplitude of 8 mA and a pulse-width of 100 ms at an interval of 2.7 s. Figure 2a and c illustrate 15 s of baseline activity for each case whereas Fig. 2b and d illustrate 15 s of their corresponding post-pacing activity. The signal traces from top to bottom were extracted from electrodes laid out from left to right in the electrode array where the pacing electrodes were located at the center of the array closest to the middle signal trace. Therefore, the baseline signals for both cases illustrate slow wave activity propagating in the antegrade direction. The electrode closest to the pacing site has been identified with a yellow star and the initiation of a pacemaker propagation pattern (earliest activation) adjacent to the pacing electrode was confirmed as the earliest activation occurs closest to this pacing electrode (see Fig. 2b and d). Pacemaker patterns in the small intestine are relatively rare based on previous spatial mapping studies, with a prevalence of around 5%^45^. Hence, the presence of a pacemaker propagation pattern centered around the pacing electrodes immediately after pacing indicates entrainment due to the pacing events.

**Figure 3:**
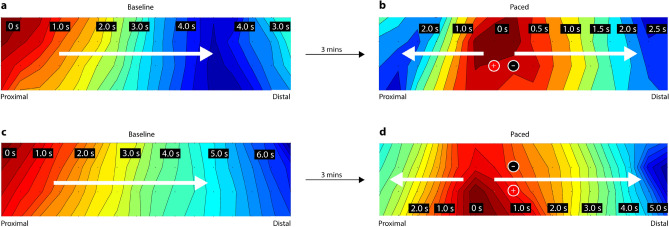
The isochronal maps corresponding to the signal traces illustrated in Fig. 2. The baseline activity in (**a**) and (**c)** propagated along the antegrade direction as indicated by the white arrows. Post-pacing activity (**b** and **d**) illustrates pacemaker propagation patterns initiated adjacent to the pacing electrode.

The propagation patterns of slow wave activity corresponding to Fig. 2 are visualized using isochronal color maps in Fig. 3. Consecutive isochronal levels depict propagation within an interval of 0.25 s where the earliest AT are indicated by dark red, and the latest AT are represented by dark blue. The white arrows indicate the propagation direction of the wave, and the location and polarity of the pacing electrodes are indicated by the ‘+’ and ‘−’ symbols. Similar propagation of slow wave activity in the antegrade direction was observed during the baseline segment for both the cases. However, the pacemaker pattern initiated in response to antegrade pacing (Fig. 3b) originated slightly above the pacing electrodes whereas the pacemaker propagation pattern initiated in response to circumferential pacing originated slightly proximal to the pacing electrodes (see Fig. 3b).

The baseline activity propagated at a similar velocity for the 2 cases shown as illustrated in Fig. 3a and c. The pacemaker pattern induced in response to antegrade pacing propagated faster than the pacemaker pattern induced in response to circumferential pacing, i.e., the dark red isochrones were larger in Fig. 3b compared to Fig. 3d.

### Optimal pacing energy

The effect of the electrical pulses applied during pacing to initiate a pacemaker pattern and achieve spatial entrainment was evaluated by comparing the pulse intensity (see Eq. 4) applied on the tissue site against the success.

**Figure 4:**
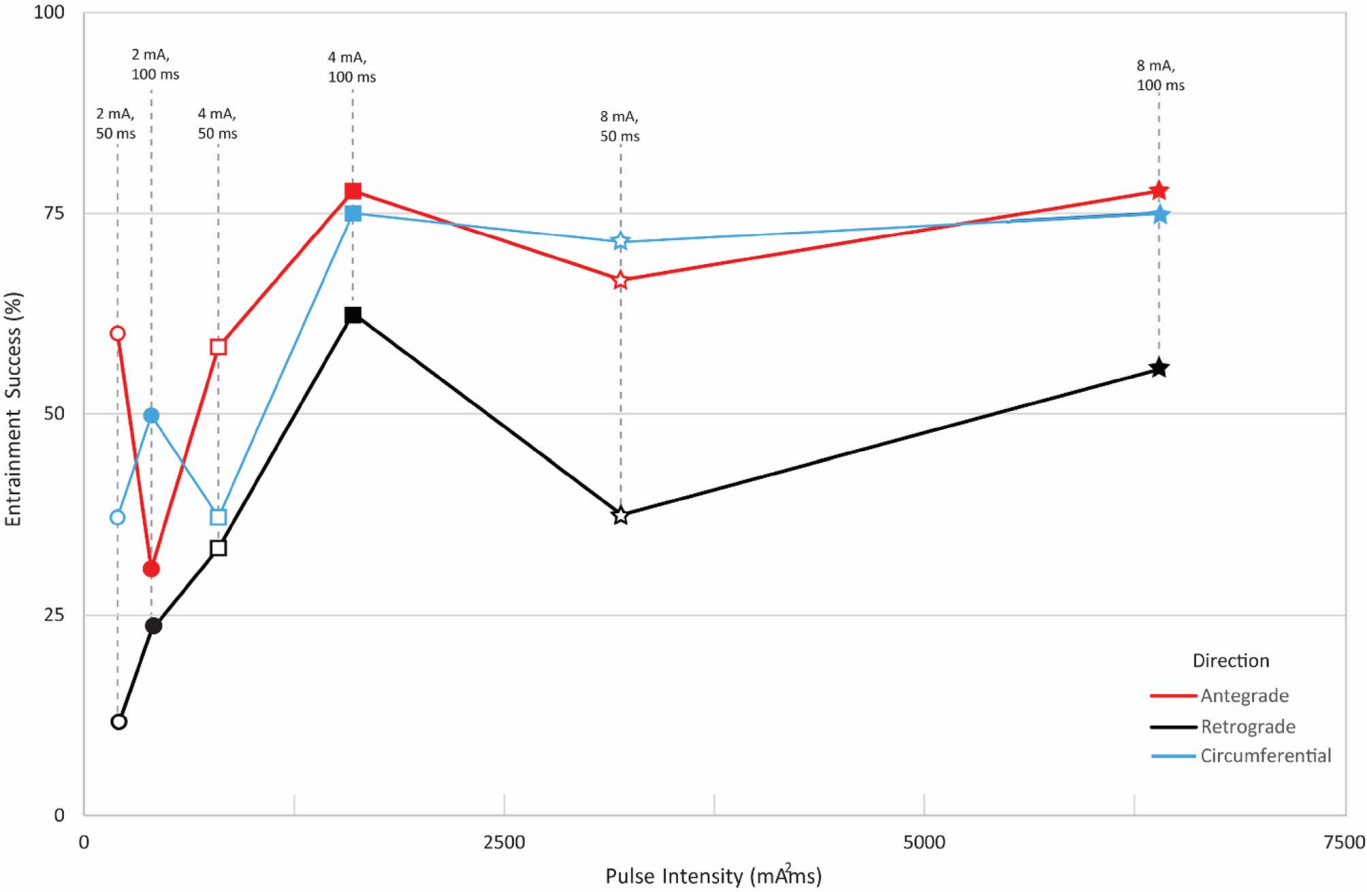
Success rates in spatially entraining slow waves of the small intestine through pacing based on the applied pulse intensity (a total of 169 recordings from 15 female pigs). The orientation of the pacing electrodes is represented by the color (antegrade = red, retrograde = black, and circumferential = blue). Pulse-amplitudes are represented by the shape (2 mA = circle, 4 mA = square, and 8 mA = star). The pulse-width is represented by the fill of the shape (50 ms = hollow, and 100 ms = solid).

**Figure 5:**
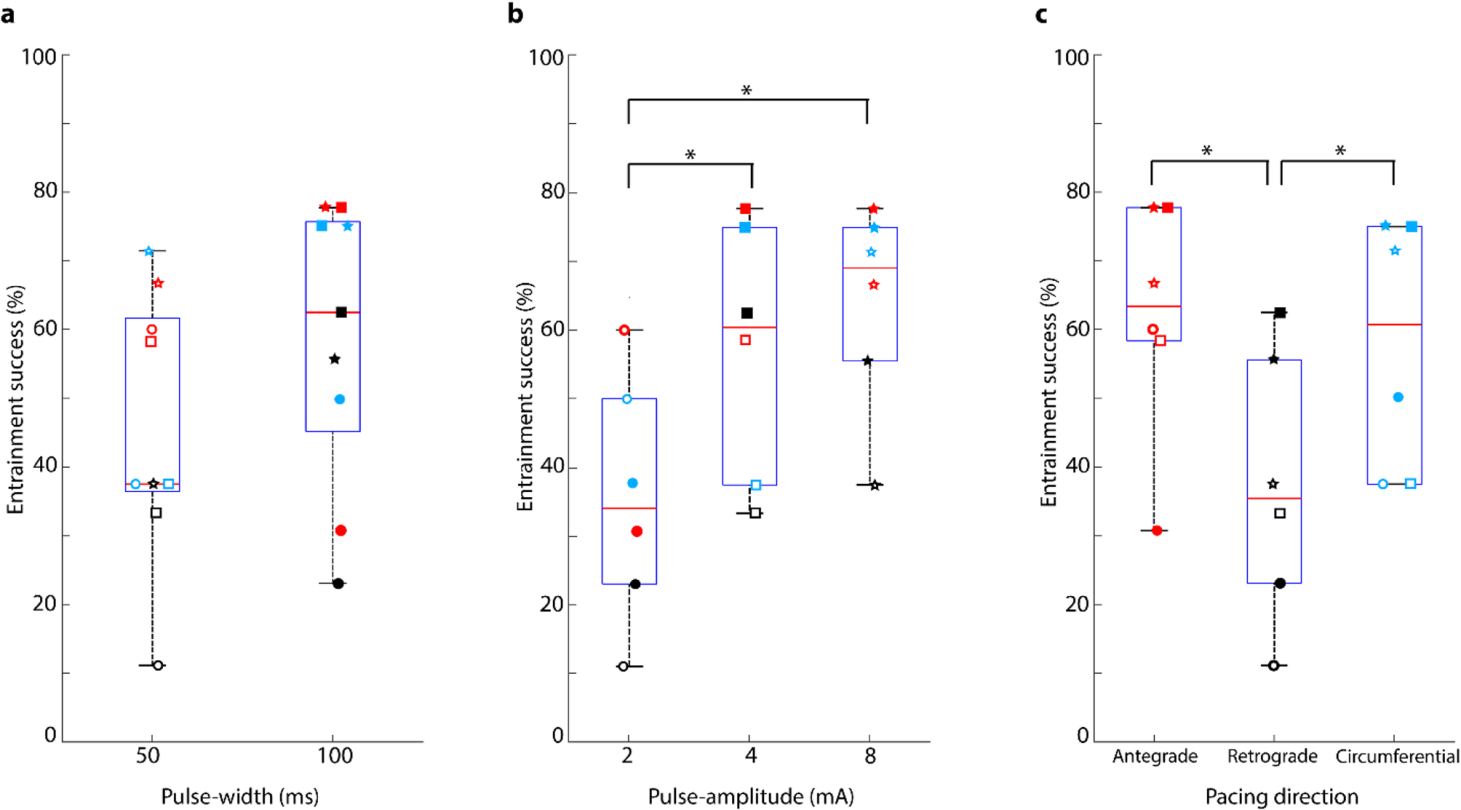
The effect of pacing (**a**) pulse-width, (**b**) pulse-amplitude, and (**c**) direction on the entrainment success rate. The orientation of the pacing electrodes is represented by the color (antegrade = red, retrograde = black, and circumferential = blue). Pulse-amplitudes are represented by the shape (2 mA = circle, 4 mA = square, and 8 mA = star). The pulse-width is represented by the fill of the shape (50 ms = hollow, and 100 ms = solid). *Indicates the statistical significance between groups based on three-way analysis of variance.

Figure 4 illustrates the percentage of success in initiating a pacemaker propagation pattern and achieving spatial entrainment for each pacing orientation with varying levels of pulse intensity. Each line on the graph depicts the outcomes for a separate pacing orientation, and the pulse parameters of each data point are labelled at their corresponding pulse intensity. The same information is presented in a different form in Fig. 5 with data grouped to highlight the influence of the pacing pulse-width (Fig. 5a), pulse-amplitude (Fig. 5b) and pacing direction (Fig. 5c) on the pacing response. The statistical significances indicated in Fig. 5 are based on three-way ANOVA.

The ANOVA results indicated that three pacing parameters (pulse-width, pulse-amplitude and pacing direction) had significant effects on the rate of spatial entrainment (*p* = 0.001, n = 169 pacing studies) while the interaction between the three pacing parameters was not significant. Pacing in the antegrade (n = 62 pacing studies) and circumferential (n = 51 pacing studies) directions achieved comparable success in spatially entraining the intrinsic activity (*p* = 0.746) and the percentage of success remained over 65.0% for all pulse intensities above 1600 mA^2^ms. Both antegrade (77.8%) and circumferential (75.0%) pacing achieved identical success with amplitudes of 4 mA and 8 mA and a pulse-width of 100 ms.

Spatial entrainment was least reliable in the retrograde direction for a pulse-amplitude of 2 mA with either pulse-width of 50 ms (11.1%) or 100 ms (23.1%). The highest reliability for retrograde pacing was 62.5% and it was achieved with a pulse-amplitude of 4 mA and a pulse-width of 100 ms. The success for retrograde pacing at the highest pulse intensity (8 mA, 100 ms) was 55.6%. While reliability in achieving entrainment in the retrograde direction improved from a pulse input of 200 mA^2^ms to 1600 mA^2^ms based on Fig. 4, the overall success for retrograde pacing remained below 63.0% for all pulse settings. Therefore, both antegrade (*p* = 0.008) and circumferential (*p* = 0.025) orientations were significantly more effective in spatially entraining the intrinsic activity than retrograde (n = 56 pacing studies) direction.

For all pacing orientations, pulse-amplitudes of 4 mA (*p* = 0.012, n = 54 pacing studies) and 8 mA (*p* = 0.002, n = 50 pacing studies) were significantly more effective in spatially entraining intrinsic slow waves compared to 2 mA for either pulse-width (50 ms or 100 ms, n = 65 pacing studies). However, there was no significant difference between 4 mA and 8 mA (*p* = 0.479) pulse-amplitudes in achieving entrainment in all pacing directions for either pulse-width. When evaluating the effect of pulse-width on the pacing response, the slow wave activity was more reliably entrained with a pulse-width of 100 ms (n = 89 pacing studies) compared to 50 ms (n = 80 pacing studies) except for one case (2 mA pacing in the antegrade direction), as depicted in Fig. 4. However, this difference was not statistically significant (*p* = 0.125).

An average tissue resistance of 1.3 ± 0.4 kΩ (n = 28 pacing sites) was measured when paced at 0.37 Hz (period of 2.7 s). Pacing was applied for a duration of 2 min at each setting and therefore, by multiplying the pulse intensity with a scaling factor of 57.78 kΩ (see Eq. 5), the total energy corresponding to each pulse intensity can be determined.

### Histological findings

Histological analysis was conducted on a total of 16 tissue sections excised from proximal jejunum of pigs (n = 10). Mean voltages of 6.0 ± 1.6 V and 7.9 ± 0.7 V were measured from tissue segments paced with a pulse-width of 100 ms and amplitudes of 4 mA and 8 mA, respectively. Therefore, the mean tissue resistance across the pacing sites was between 1.0 ± 0.1 kΩ and 1.5 ± 0.4 kΩ and the mean energy applied for each 10-min pacing session was between 0.2 ± 0.1 J and 0.5 ± 0.1 J for a pulse-amplitude of 4 mA and 8 mA.

**Figure 6:**
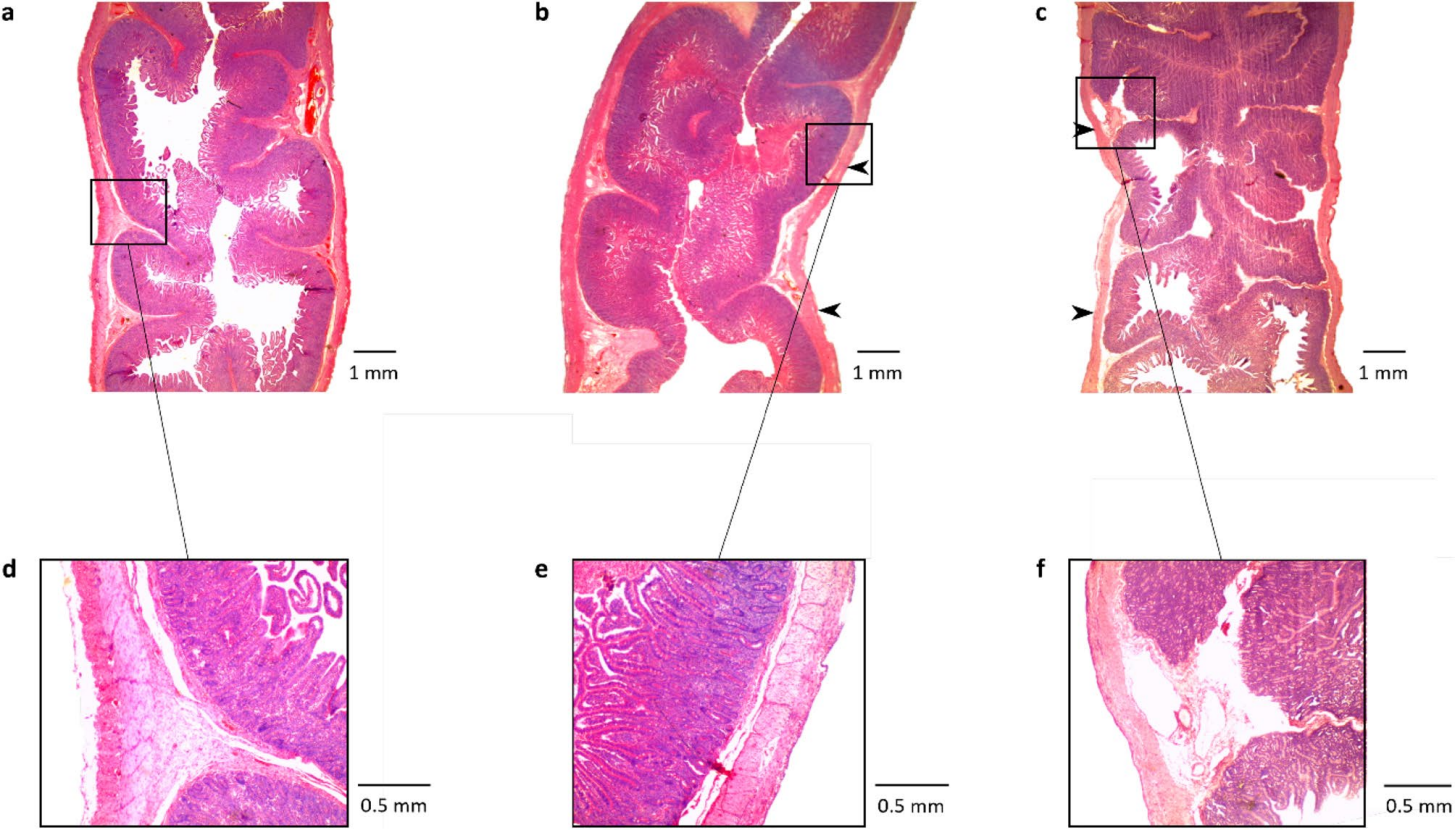
Histological comparison of hematoxylin and eosin stained (**a**) control and (**b**–**c**) paced tissue slices of the jejunum. The segments were paced with a pulse-width of 100 ms and pulse-amplitudes of (**b**) 4 mA and (**c**) 8 mA. The locations of pacing sites on the tissue slices are indicated using black arrows. Enlarged views of the areas highlighted using black squares in (**a**–**c**) are illustrated in (**d**–**f**), in order.

The structure of all tissue slices was visually examined under a microscope and compared against control tissue slices to assess for tissue damage. Signs of tissue damage, such as deformed tissue structures, enlarged nuclei or congested blood vessels were not observed. As no tissue damage was observed in the first 16 tissue samples, it was concluded that the pacing settings investigated in this study do not cause tissue damage, and histological analysis was not performed in the subsequent studies. Figure 6 illustrates representative control (Fig. 6a) and paced tissue slices (Fig. 6b,c) with enlarged views of the pacing sites (Fig. 6e,f) and control tissue (Fig. 6d). No signs of tissue damage are evident.

## Discussion

The efficacy of pacing parameters including pulse-amplitude, pulse-width and pacing direction was systematically investigated to determine optimal pacing settings for entraining slow wave activity in the small intestine for the first time. A novel high-resolution framework previously validated for spatial entrainment of the jejunum was adopted in this study^33^. While our previous study reported initial results on the response to pacing the jejunum with only 2 energy levels, this study systematically varied pulse-amplitude from 2 to 8 mA and pulse-width from 50 to 100 ms in the antegrade, retrograde and circumferential directions.

As intrinsic pacemaker propagation patterns are infrequent and observed only 5% of the time in mapping studies, the fact that such patterns initiated in 77% of cases (4 mA and 8 mA) immediately after pacing indicates that pacing has successfully modulated the slow wave activity. In order to determine the optimal pacing setting for spatial entrainment, the pulse intensity was computed and the percentage of success in achieving spatial entrainment at these intensities was compared against different pacing directions. The pulse intensity allows comparison solely based on pulse inputs as opposed to energy which accounts for tissue resistance. Antegrade and circumferential pacing performed comparably and the highest percentage of success for both directions was identical with a pulse-width of 100 ms and pulse-amplitudes of 4 mA and 8 mA. However, retrograde pacing was inferior to both antegrade (*p* = 0.008) and circumferential (*p* = 0.025) pacing. As the intrinsic dominant frequency of the interstitial cells of Cajal in the small intestine decreases along the length of the intestine^46^, this frequency gradient likely causes the paced activity to have a preferential propagation in the antegrade direction as opposed to the retrograde direction.

Based on Fig. 4, the entrainment was reliable for pulse intensities beyond 1600 mA^2^ms and remained stable thereafter. Therefore, pacing in the antegrade or circumferential directions with a pulse-amplitude of 4 mA and a pulse-width of 100 ms can be considered optimal as this pulse intensity achieves the highest success in achieving spatial entrainment with minimum energy. Ensuring reliable entrainment with minimal energy is crucial for preserving the battery life of a chronic implant. Furthermore, excessively high energy levels are more likely to result in tissue damage and may directly trigger smooth muscle contractions rather than the targeted pacemaker cells^47^, whereas higher frequency stimulation has been shown to trigger neural pathways^4^. Therefore, the key to long term pacing lies in achieving reliable entrainment to modulate pacemaker cells in the gastrointestinal tract, commonly referred to as interstitial cells of Cajal, while simultaneously minimizing energy consumption.

Pre-clinical gastric pacing studies have previously identified minimum pulse intensities between 400 mA^2^ms and 12,500 mA^2^ms to be necessary for slow wave entrainment^29,32,48^. However, only one group of studies achieved entrainment using a pulse intensity less than 1600 mA^2^ms^29,49^. While our study was able to spatially entrain with a pulse intensity of 400 mA^2^ms (2 mA, 100 ms), 1600 mA^2^ms was found to provide more consistent results. Therefore, the optimal pulse intensity for small intestine pacing is less than the intensity defined by most gastric pacing studies. The thickness of the gastric wall is greater than that of the small intestine and therefore, the serosal layer of the small intestine is closer to the pacemaker cells (i.e., the interstitial cells of Cajal) than in the stomach^50,51^. This indicates that the energy is likely to be more readily dissipated from the serosa to the pacemaker cells in the small intestine compared to the stomach and that this may be a plausible explanation for the lower pulse intensity. However, both this study and the previous gastric studies which achieved entrainment with 400 mA^2^ms were performed using surface-contact electrodes^29,49^, and the larger electrode contact surface may be a feature unique to surface-contact pacing over other types of pacing electrodes.

When paced with a pulse-amplitude of 2 mA, the percentage of success in achieving spatial entrainment was inconsistent and highly variable when paced in the any direction with either a pulse-width of 50 ms or 100 ms (see Fig. 4). However, amplitudes of 4 mA and 8 mA were significantly more effective in achieving entrainment compared to 2 mA. Previous small intestinal pacing studies have determined a pulse-amplitude of 4 mA to be optimal whereas the optimal pulse-width was variable^52–54^. There was no significant difference between the applied pulse-widths in this study and 100 ms was determined optimal based on the optimal pulse intensity, i.e., 1600 mA^2^ms. Lin et al.^52^, reported a pulse-amplitude of 140 ms to be optimal in pacing the jejunum, whereas pulse-widths of 160 ms and 10–20 ms have been reported optimal for duodenum^53,54^. However, none of these studies considered the pacing direction and the optimal settings were based on the assessment of temporal changes in slow wave frequency assessed by a few sparse electrodes. Previous investigations used limited number of electrodes that do not provide information on the propagation patterns whereas high-resolution techniques used in this study allow both spatial and temporal propagation information. Nevertheless, the optimal pulse-amplitude agrees with previous reports and the pulse-width determined is less than the pulse-width reported by Lin et al., in pacing the jejunum.

Histological analyses were conducted for tissues paced with 1600 mA^2^ms and 6400 mA^2^ms pulse intensities which corresponded to the energy levels with highest percentages of success. However, no tissue damage was observed from any of the tissue specimens. Only a single small intestinal pacing study has previously conducted histological analysis of the pacing sites and has reported scarring of the serosa possibly from suturing the electrode to the bowel^24^. However, they also applied comparatively higher pulse-amplitudes 25–30 mA. Future research can improve tissue assessment by exploring tissue inflammation with histological markers like the Geboes score and blood biomarkers, and evaluating cellular stress by examining DNA and RNA damage^55,56^. Studies conducted on the stomach using surface-contact electrodes have also reported success in pacing without damaging the stomach wall^29^, which suggests surface-contact pacing to be a safe technique at the applied energy levels.

A key limitation of this study is the acute setting which does not enable the evaluation of the long-term effect of pacing. In addition, functional changes such as transit rate of luminal contents were not examined in this study. The analysis of the pacing response was restricted to post-pacing activity as the activity induced during pacing was obstructed by the onset of periodic stimuli. The small intestine pacing frequency is faster than the gastric pacing frequency and therefore, the activity in between pacing pulses does not allow sufficient time for the complete propagation of wavefronts. Furthermore, surgery, visceral handling, and anesthetics, as used in this study, have been shown to influence slow wave activity^57^, but numerous studies have successfully investigated normal slow wave behavior in different anesthetized animal models^58–60^.

Future research will seek to define the long-term effects of small intestine pacing based on chronic studies. Development of micro-electrode arrays and miniaturized devices suitable for implantation on the small intestine is necessary to conduct chronic studies. While a few studies have successfully normalized dysrhythmic slow wave activity temporally by pacing^22,61,62^, dysrhythmic slow wave activity is yet to be spatially entrained in the small intestine by pacing. It is hypothesized that multi-point pacing requires less overall energy to entrain slow waves over an extended length of the bowel, and this can be investigated in the future. In addition, the role played by small intestine pacing on motility functions can be explored by mapping the contractile response. Closed-loop pacing strategies which minimize battery consumption can also improve the efficacy of this promising technique^63,64^.

## Conclusion

Optimal pacing parameters to reliably achieve spatial entrainment of slow waves in the small intestine were determined. By varying the pacing parameters systematically, pulse intensities below 1600 mA^2^ms (pulse-amplitude of 4 mA and pulse-width of 100 ms) were found to be less effective in entraining slow wave activity. Pulse-amplitudes greater than 2 mA were found to be more effective than pulse-widths of 50 ms or 100 ms. Therefore, a pulse-amplitude of 4 mA and pulse-width of 100 ms were defined as optimal for slow wave entrainment while also minimizing energy input, when paced in the antegrade or circumferential direction of the jejunum. Future studies can utilize the optimal pacing configurations to assess the long-term effectiveness of the pacing response and examine functional changes such as changes in emptying rate and body weight.

## Data Availability

The data that support the findings of this study are available from the corresponding author upon reasonable request.
